# Screening for juvenile idiopathic arthritis associated uveitis with laser flare photometry in the pediatric rheumatology office: a prospective observational study

**DOI:** 10.1186/s12969-024-00961-9

**Published:** 2024-01-26

**Authors:** Kaleo Ede, Michael Shishov, Elisa Wershba, Nikita Goswami, Sabrina Gorry, Malin Joseph, Lucia Mirea, James O’Neil

**Affiliations:** 1 Division of Pediatric Rheumatology, Phoenix Children’s Hospital, 1919 E Thomas Rd, 85016 Phoenix, AZ USA; 2 Department of Biostatistics, Phoenix Children’s Hospital, 1919 E Thomas Rd, 85016 Phoenix, AZ USA; 3 Division of Pediatric Ophthalmology, Phoenix Children’s Hospital, 1919 E Thomas Rd, 85016 Phoenix, AZ USA

**Keywords:** Juvenile idiopathic arthritis asociated Uveitis, Laser flare photometry, Screening, Diagnosis

## Abstract

**Background:**

Juvenile Idiopathic Arthritis (JIA) Associated Uveitis (JIA-U) remains one of the most serious complications of JIA in children. Historically, pediatric JIA is diagnosed by an Optometrist or Ophthalmologist; however, barriers to scheduling increase wait times that may delay diagnosis and treatment. The purpose of this study was to evaluate laser flare photometry (LFP) use to diagnose JIA-U in the Pediatric Rheumatology clinic for patients with JIA.

**Methods:**

This prospective, observational study assessed pediatric patients diagnosed with JIA without a previous history of uveitis between January 2020 and September 2022. All patients underwent at least one evaluation of both eyes using a Kowa FM-600 laser flare photometer during a routine Rheumatology appointment, as well as a standard slit lamp examination (SLE) by optometry or ophthalmology during routine clinical care. Data collected at patient visits included demographics, JIA characteristics, treatment, LFP readings, and anterior chamber (AC) cell grade score utilizing the SUN grading system. Data were summarized using descriptive analyses and the uveitis false positive rate was calculated.

**Results:**

The study cohort included 58 pediatric patients diagnosed with JIA. The mean age was 8.4 years (1.2–16.3 years) at diagnosis and 11.9 (4.8–16.5 years) at enrollment. The mean duration of disease at time of enrollment was 42 months (range; 0-157 months). Participants were predominantly female (*n* = 43, 74.1%) and white/Caucasian race (*n* = 37, 63.8%). The most common JIA subtypes included persistent oligoarticular JIA (*n* = 19, 32.8%), and RF negative polyarticular JIA (*n* = 12, 20.7%). There were 12 ANA positive patients (20.7%). At enrollment, 16 patients (27.6%) were not on medications, with 20 (34.5%) on methotrexate, 20 (34.5%) on adalimumab, 6 (10.3%) on tocilizumab, and 5 (8.6%) on etanercept. During the study period, no eye exams detected active uveitis based on SLE with a SUN grade over 0. However, of the 135 LFP readings, 131 (97.0%) were normal, yielding a false positive rate of 3% (95% CI: 0.8%, 7.4%).

**Conclusions:**

LFP is a non-invasive tool that can be utilized in the pediatric rheumatology clinic to evaluate for JIA-U. There is a low false positive rate of LFP when compared with standard slit lamp exam.

## Background

Juvenile Idiopathic Arthritis (JIA) is the most common chronic rheumatic disease in childhood, affecting between 1 and 22 per 100,000 children in 2022 [1]. JIA is a heterogenous group of autoimmune diseases characterized by the presence of arthritis in at least one joint for a duration of 6 weeks or more that begins before 16 years of age [2, 3]. The most frequent extra-articular manifestation of JIA is JIA-associated uveitis (JIA-U), a chronic non-granulomatous inflammation of the anterior chamber of the eye [4]. The prevalence of JIA-U is between 11 and 30% of all JIA patients and can lead to sight-threatening complications including synechiae, cataracts, glaucoma, and vision loss [5]. JIA-U most often occurs within the first 4 years of onset of arthritis, although up to 12% of patients can present with uveitis before the onset of arthritis [6]. In the United States, there is a median delay in diagnosis of 8 months (range 0–16 years) for patients with JIA-U. The primary reason for delayed diagnosis is the absence of linked symptoms, compounded by the fact that uveitis may precede the onset of symptoms in as much as 12% of cases [7]. 

The current gold standard tools used to screen for JIA-U include age-appropriate visual acuity (VA) testing, measurement of intraocular pressure and slit lamp examination (SLE) [5]. The diagnosis of uveitis relies on identifying features of inflammation upon SLE. These features encompass the presence of cells in the anterior chamber (AC) and AC flare, leading to protein leakage into the AC due to the breakdown of the blood-aqueous humour barrier [8]. Intraocular inflammation is manually graded according to the Standardization of Uveitis Nomenclature (SUN) criteria [9]. This requires all JIA patients to be seen by ophthalmologists or optometrists on a regular schedule depending on the presence of disease specific risk factors.

Laser flare photometry (LFP) is an instrument that quantifies scattered light resulting from a laser aimed at the anterior chamber. The purpose of this measurement is to estimate the quantity and size of proteins present in the aqueous humour, with a large scale of values ranging from 4 photons per millisecond (ph/ms) in non-inflamed eyes to values as high as 1000 ph/ms in the presence of inflammation [10]. Recently, LFP has been shown to have a predictive value in monitoring the course and complications of pediatric non-infectious uveitis, as well as to stratify risk of long-term complications in patients with JIA-U [11, 12]. While LFP has been used in research and clinical practice by ophthalmologists, there is no current literature documenting the utility of LFP in the pediatric rheumatology outpatient setting. The purpose of this study was to determine if LFP is a useful tool in the pediatric rheumatology office to diagnose patients with JIA-U.

## Methods

### Subjects and data collection

**Fig. 1:**
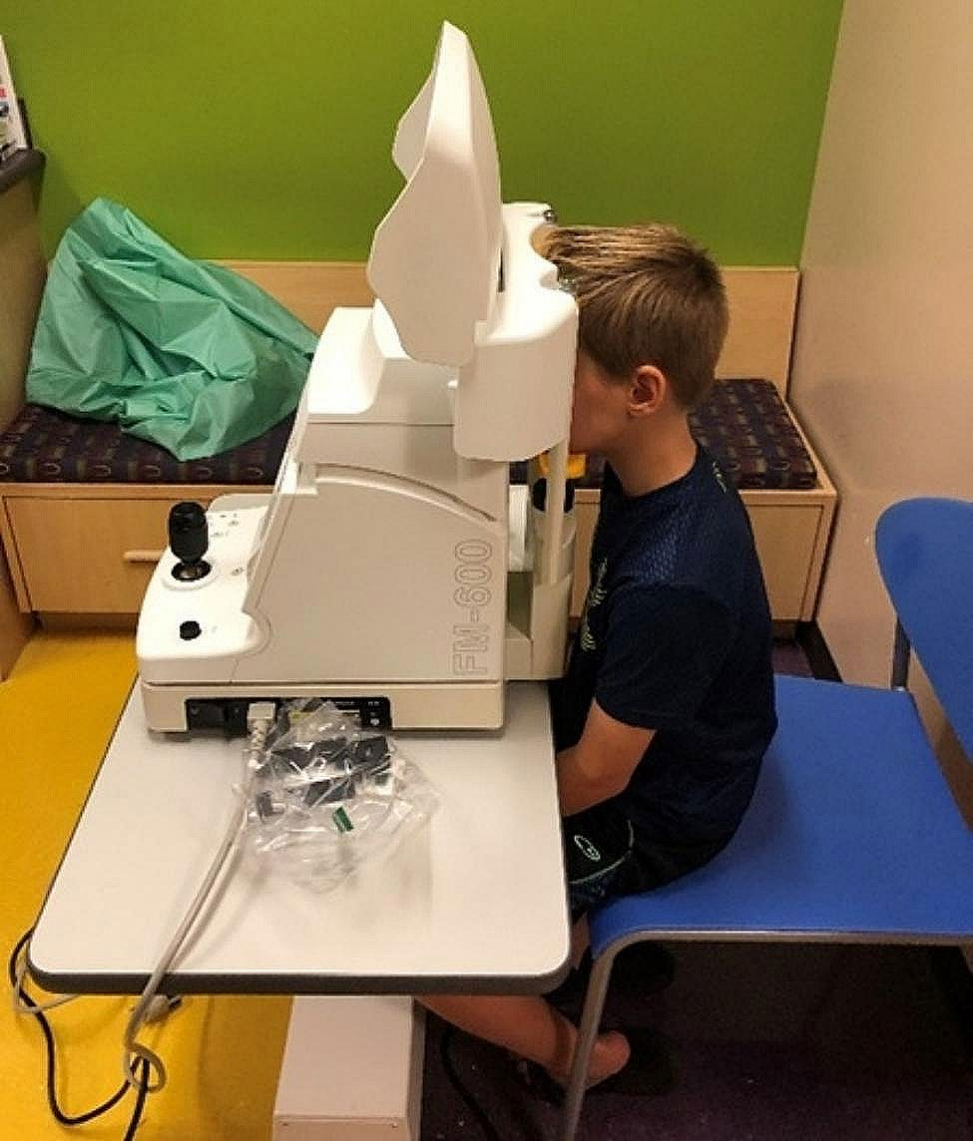
Patient undergoing LFP exam with Kowa FM-600

This prospective, observational study was conducted at Phoenix Children’s Hospital from January 2020 through September2022 and approved by the Phoenix Children’s IRB (IRB-19-570). Patients aged 4–16 years diagnosed with JIA based on the 2001 International League of Associations for Rheumatology classification criteria were included in the study [13]. Exclusion criteria included a history of JIA-associated uveitis or any other form of uveitis, as well as history of pacemaker placement. All patients underwent at least one evaluation of both eyes using a Kowa FM-600 laser flare photometer (Kowa Company, Ltd., Electronics and Optics Division, Tokyo, Japan) (Fig. 1). Additionally, all patients had standard SLE performed by an Optometrist or Ophthalmologist during routine clinical care. Data collected at patient visits included demographics (age at diagnosis, age at enrollment, sex and race), JIA subtype, treatment medications, LFP readings, and AC cell grade scored by the SUN grading system.

### Statistical analysis

Descriptive analyses were utilized to summarize the data, and the false positive rate and corresponding 95% confidence interval was calculated.

## Results

A total of 58 children diagnosed with JIA participated in this study and data were collected from 69 visits (Table 1). The mean age at diagnosis was 8.4 years (range; 1.2–16.3 years) and 11.9 years at enrollment (range; 4.8–16.5 years). The mean duration of disease at time of enrollment was 42 months (range; 0-157 months). Of the 58 patients, the majority (74.1%, *n* = 43) were female, and most belonged to the white/Caucasian racial group (63.8%, *n* = 37).

**Table 1:** Patient demographics

Characteristic	Overall (*N* = 58 patients)
**Sex**	
Female Male	43 (74.1%) 15 (25.8%)
**Race**	
Asian Black/African American Hispanic/Latino Native American White/Caucasian Other	2 (3.4%) 2 (3.4%) 15 (25.9%) 1 (1.7%) 37 (63.8%) 1 (1.7%)
**Age at diagnosis (years)**	
Mean (SD) Median Q1, Q3 Range	8.4 (4.2) 8.6 4.9, 12.2 (1.2, 16.3)
**Age at enrollment (years)**	
Mean (SD) Median Q1, Q3 Range	11.9 (3.2) 12.4 9.9, 14.6 (4.8, 16.5)
**Duration of disease at enrollment (months)**	
Mean (SD) Median Q1, Q3 Range	42.0 (40.2) 34.0 12.0, 59.0 (0, 157.0)

The predominant JIA subtypes comprised 19 (32.8%) patients with persistent oligoarticular JIA, and 12 (20.7%) with Rheumatoid Factor (RF) negative polyarticular JIA. There were 12 patients (20.7%) that were ANA positive. Upon enrollment, 16 patients (27.6%) were on no medications as they were in remission, while 20 (34.5%) were being treated with methotrexate, 20 (34.5%) were on adalimumab, 6 (10.3%) were receiving tocilizumab, and 5 (8.6%) were undergoing treatment with etanercept (Table 2). There were 9 (15.5%) patients on combination biologic and methotrexate therapy, and 22 (37.9%) on biologic therapies alone. During the study period, no eye exams detected active uveitis based on slit lamp exam with a SUN grade over zero. However, of the 135 LFP readings, 131 (97.0%) were normal, yielding a false positive rate of 3% (95% CI of 0.8%, 7.4%) (Table 3).

**Table 2:** Patient disease characteristics and treatments

Characteristic	Overall (*N* = 58 patients)
**JIA subtype**	
Oligoarticular RF (-) Polyarticular RF (+) Polyarticular Systemic Enthesitis-related arthritis Psoriatic Undifferentiated	19 (32.8%) 12 (20.7%) 8 (13.8%) 3 (5.2%) 10 (17.2%) 4 (6.9%) 2 (3.4%)
**Lab testing**	
ANA positivity	12 (20.7%)
**Treatment at baseline**	
Methotrexate Adalimumab Etanercept Tocilizumab Other biologic None	20 (34.5%) 20 (34.5%) 5 (8.6%) 6 (10.3%) 0 16 (27.6%)

**Table 3:** Comparing SLE to LFP results

Slit lamp result or equivalence	Slit lamp exam *N* = 138	LFP exam *N* = 135 ^a^
**Normal** (0.5 to 9 LFP flare)	138 100.0%	131 97.0%
**Low amount of flare** (10 to 25 LFP flare)	0 0.0%	1 0.7%
**1 + to 2- slit lamp reading** (76 to 125 LFP Flare)	0 0.0%	3 2.2%
**Total false positive rate** N, % (95% CI)	--	4 3.0% (0.8%, 7.4%)

^a^ 3 patients only had one eye measurement taken at rheumatology visit

^b^ A false positive is defined as a normal result on the slit lamp exam and anything other than normal result on the LFP exam

## Discussion

This is the first prospective study to assess the use of LFP in the pediatric rheumatology clinic to evaluate for the presence of occular inflammation in patients with JIA. We identified agreement between LFP values and SLE findings performed by pediatric ophthalmologists, with a low false positive rate of 3%. This suggests that LFP may be a useful tool in clinical practice to screen for JIA-U.

There is a high rate of occular complications and vision loss in patients with JIA-U, with an overall complication rate of 0.33 events per eye-year and a rate of visual acuity loss to 20/50 or worse of 0.1 events per eye-year [14]. A recent report highlights the importance of accurate and objective tools to compliment the standard SLE by ophthalmologists in order to avoid these long term complicaitons [15]. LFP has increasingly been used by opthalmologists in this context. Maccora et al. conducted a monocentric Italian study, detailing their experience with LFP in monitoring childhood chronic uveitis. Noteworthy was the correlation observed between LFP measurements and SLE findings. Furthermore, a positive association was identified between elevated LFP values and the presence of ocular complications [16]. Additionally, LFP has been used in a double-blind, randomised, placebo-controlled trial of adalimumab in early onset JIA-U patients as a primary outcome measure in addition to SLE [17]. Combined with the results of our current study, this furthers the notion that LFP can be a complimentary tool for pedaitric rheumatologsits to aid in the detection and management of JIA-U.

This study was limited by the small number of participants resulting from a low enrollment rate which was significantly affected by the coronovirus disease 2019 (COVID-19) pandemic. Additionally, our incidence of new onset JIA-U was lower than expeceted, as we did not identify any patients with JIA-U in our population. Future directions of LFP studies in the pediatric rheumatology clinic should include patients with existing JIA-U, as well as patients with other forms of chronic anterior uveitis to determine if it is a useful tool to monitor for treatment outcomes and the development of ocular complications.

## Conclusions

This work demonstrates the feasibility of using LFP in the pediatric rheumatology clinic to screen for uveitis in patients with JIA. LFP represents a potentially easy-to-use tool to compliment formal opthalmologic SLE and may eventually aid in decreasing ocular complications from JIA-U. The ability to systematically screen patients for JIA-U within the rheumatology clinic holds the potential to unveil signs at their earliest stages, a crucial stride toward not only timely detection but also the prospect of preventing vision loss in JIA patients.

## Data Availability

The raw data supporting the conclusions of this study will be made available by the authors, without undue reservation.

## Abbreviations

JIA: Juvenile Idiopathic Arthritis
JIA-U: Juvenile Idiopathic Arthritis associated uveitis
LFP: Laser Flare Photometry
SLE: Slit Lamp Exam
AC: Anterior Chamber
SUN: Standardization of Uveitis Nomenclature
RF: Rheumatoid Factor
COVID-19: Coronavirus disease 2019
